# Synthesis, Characterization and Performance of Materials for a Sustainable Future

**DOI:** 10.3390/polym16010124

**Published:** 2023-12-29

**Authors:** John Vakros, Evroula Hapeshi, Catia Cannilla, Giuseppe Bonura

**Affiliations:** 1 Department of Chemical Engineering, University of Patras, Caratheodory 1, University Campus, 26504 Patras, Greece; vakros@chemistry.upatras.gr; 2 Department of Health Sciences, School of Life and Health Sciences, University of Nicosia, 46 Makedonitissas Avenue, CY-2417, P.O. Box 24005, Nicosia 1700, Cyprus; hapeshis.e@unic.ac.cy; 3 Institute for Advanced Energy Technologies “Nicola Giordano” ITAE, National Research Council (CNR), 98126 Messina, Italy; catia.cannilla@cnr.it

The current era has been defined as “The Plastic Era”, considering that over the past 50 years the role and importance of polymeric materials in our economy has steadily grown, reaching a production of around a few hundred million tons per year which may even double in the next 20 years. There is clear evidence that the use of fossil-based substances for the production of plastic materials will lead to progressive air pollution and an increase in global temperature. The establishment of alternative and more sustainable production chains for plastics cannot be postponed any longer due to the important role that plastics play in society and in the e-economy as long as they are intelligently and responsibly used, managed and recycled. Herein, not only polymers in the narrow sense, but also their hybridizations as green composite materials for novel applications in existing or emerging sectors penetrating the market, are used to highlight the breakthroughs in the transition to bio-plastics that meet the principles of circular economy.

Bio-based polyurethane formulations, synthesized by combining Organosolv lignin and a commercial isocyanate, were indicated to be promising polymers with suitable properties for the coating industry [1]. Additives such as film-formers with low surface tension to counteract crack formation were found to be necessary to improve the performance of lignin-based coatings. The lignin concentration also affected the general appearance of the coatings, which presented many cracks for higher lignin contents.

An interesting feature demonstrated by Yan et al. [2] is the acoustic performance and flame retardancy exhibited by ammonium polyphosphate/diethyl ethylphosphonate rigid polyurethane foams synthesized using a one-pot free-rising method. In particular, the addition of ammonium polyphosphate was shown to improve the acoustic absorption performance of the foam, as verified by acoustic absorption measurements. Considering its potential applications, a balanced concentration of ammonium polyphosphate (APP) and diethyl ethylphosphonate (DEEP) was proposed to be effective for the preparation of a new flame-retardant acoustic absorption rigid polyurethane foam. Indeed, APP allowed the foam matrix to form an intumescent carbon layer which protected the foam from further decomposition. Meanwhile, DEEP released highly active species to capture free radicals produced by the foam matrix, leading to a rapid self-extinguishing effect. 

Among the critical issues preventing the market penetration of bio-composites is the interfacial bonding between natural fibers and polymer matrices. In this regard, Alzebdeh et al. [3] described a new synthesis route comprising the treatment and functionalization of both date palm powder (DPP) filler and a polypropylene (PP) matrix to enhance filler–polymer adhesion in newly developed bio-composites. Fourier transform infrared spectroscopy (FTIR) confirmed the successful coupling between the filler and polypropylene matrix after applying the treatment and functionalization schemes. The applied compatibilizers assisted with reducing the water uptake of the manufactured bio-composites, increasing their durability and paving the way for applications of such functionalization techniques to other types of polymers and natural fillers requiring superior bio-compatibility. 

It is clear that the increased stability of plastic-based materials determines new issues about their resistance to degradation with serious impacts on the environment and society. To tackle this need, the response surface methodology was applied to investigate the optimal conditions, in terms of the mass ratio of catalyst to polyethylene terephthalate (PET), temperature, and time, for the glycolysis of PET with the aid of oyster shell-derived catalysts [4]. Considering the economic and environmental aspects, the optimal conditions were determined to be as follows: 195 °C, 45 min, 1 wt.% for reaction temperature, time and mass ratio of catalyst to PET.

An unconventional application of polymeric materials was also reported in the bio-medical field, considering the development of a novel adsorbent capable of adsorbing influenza viruses in the form of aerosols in the air [5]. In particular, a functional group, i.e., N-acetylneuraminic acid, was introduced into a microfiber nonwoven fabric, manufactured through radiation-induced graft polymerization (RIGP), and sialic acid was immobilized to mimic the sugar chain cluster effect. Additional experiments are expected to clarify the optimal monomer concentration for RIGP and the reaction conditions for virus capturing.

More relevant to the technological sector is the potential of geopolymer foams (GFs), as synthesized from metakaolin and silica sand, along with chopped carbon fibers and finally doped with the addition of different aluminum-rich industrial by-products. The results of Ercoli et al. [6] identified GFs with good mechanical and thermal insulation properties, encouraging future researchers to find the best combination (for types and proportions) of the different by-products of the secondary aluminum industry to produce lightweight geopolymer foams as alternative composite materials. The reuse of these industrial by-products, which according to European Regulations cannot be disposed of in landfill, raises issues related to environmental sustainability and safe management that must be properly taken into account.

The need for clean water, as disinfected from microbial contamination, is the basis of an innovative and sustainable utilization of metal-based composite reinforcements, integrating modified Ag–MgO–nanohydroxyapatite on a nanofibrous cellulose template (CNF-AgMgOnHaP). This composite acts as a multifunctional adsorbent of inorganic and microbiological hazards, and was developed via a hydrothermal bioreduction route using Citrus paradisi peel extract [7]. The synthesized composite not only exhibited a significant defluorination capacity, but also provided some antibacterial activity against common infectious microbes from contaminated drinking water. 

Another strategy arises from the use of lignin as high added value for polymer blends and composites. In particular, the chemical modification of Kraft lignin was demonstrated to be effective for blending with PP, which is a multifunctional and widely applied polymer, although characterized by a low-energy surface and poor adhesion, preventing prospective applications in composites [8]. Indeed, the lignin modifications led to a better compatibility with the PP matrix and surface energies up to 86% higher than neat PP, envisaging the development of new green adhesion methods.

Another current challenge related to the management of environmental pollution concerns the recycling and reuse of waste-printed circuit boards (WPCBs). He et al. [9] presented a method for improving the durability and mechanical properties of WPCB-reinforced polymer composites, demonstrating that their hybridization with SiO_2_ enhanced tensile strength, flexural strength and flexural modulus with respect to the unmodified system. Those properties affected the impact strength when combined in a polypropylene-based matrix, showing results 28.8% higher than its untreated counterpart owing to the serrated interface. 

Biomass-derived compounds also have the potential to be modified in order to expand their range of application. In this respect, Marseno et al. [10] reported that the hydrophilic properties of sago starch can be significantly changed by heat moisture treatment (HMT), following a clear alteration of physico-mechanical properties. In particular, pre-treatment via HMT before esterification with octenyl succinic anhydride (OSA) increased the degree of substitution, reaction efficiency and hydrophobicity of the OSA starch. 

Considering the relevant influence of functional groups on the porosity structure and adsorption efficiency of polymer materials, Alkayal et al. [11] synthesized a novel porous polyaminal-linked polymer, based on naphthalene and melamine (PAN-NA) building blocks, via a one-pot polycondensation method, in order to probe its functionality as an adsorbent for both CO_2_ and heavy metals. It was found that naphthyl, with additional active sites and increasing π-surface area features, is an excellent choice for the formation of microporous polymers, accounting for varied porosity and good performance of gas adsorption. The ability of the PAN-NA polymer in heavy metal adsorption was demonstrated by its selectivity toward the Pb(II) cation, with an adsorption capacity of 100.7 mg/g at pH = 6, 20 mg/L and an adsorbent dose of 20 mg after 20 min.

Regarding recyclable matrices for composite materials, vitrimers combine thermoset properties with reprocessability, although their mechanical performance for specific applications has not been fully demonstrated. The reinforcement of a vitrimer formulation with carbon fibers, consisting of functional epoxy groups and a new dynamic disulfide crosslink-based hardener, induced the typical requirements for high-performance structural components, comparable to those currently used in aircraft materials [12]. The dynamic properties of this formulation have to be more thoroughly investigated in order to establish a processing window in which this formulation could be reprocessed by maintaining the overall composite properties.

In terms of biocompatibility, polyacrylic acid (PAA) represents a good candidate for conventional and novel drug carrier systems. PAA and its nanoconjugates can also be regarded as stimuli-responsive platforms, making them ideal for drug delivery and antimicrobial applications [13]. Of course, merging biological and synthetic viewpoints will provide a new perspective for the development of more effective polymeric nanoplatforms. PAA nanoplatforms could have great potential for the research and development of new nanovaccines and nanodrugs in the future.

Plasticizers are an important class of compounds widely used as additives in the polymer industry to improve properties and polymer processing. Ledniowska et al. [14] synthesized four environmentally friendly plasticizer samples from renewable raw materials using succinic acid, oleic acid, and propylene glycol. The obtained ester mixtures were used as poly(vinyl chloride) (PVC) plasticizers and their plasticization efficiency was determined in comparison to traditional, commercially available toxic phthalate plasticizers. It was observed that the obtained plasticizers exhibited the same plasticization efficiency and were characterized by good mechanical and physical properties in comparison to commercial plasticizers. The ester mixture that was found to be the most favorable plasticizer was characterized by good thermal and thermo-oxidative stability (5% weight loss temperature: 227.8 °C in air and 261.1 °C in nitrogen). 

The limited availability of substances typically bonded in polymer chains raises demand for alternative bulk chemicals, as in the case of phenol-formaldehyde resins widely used in outdoor wooden products because of their strong mechanical properties, stable molding processing performance and high flame retardancy. Yu et al. [15] prepared bio-oil phenol–formaldehyde (BPF) resins by using bio-oil as a substitute for phenol, reporting similar bonding strength but lower price compared to phenol–formaldehyde (PF) resin. The comparison of data between BPF and PF resins after aging 960 h showed that adding bio-oil could obviously weaken the aging effect of water but slightly enhance that of heat. The results could provide a basis for the aging resistance properties of BPF resin.

Polyhemiaminal (PHA) polymers are another class of thermosetting polymers that have recently gained attention owing to their high mechanical strength and excellent recycling behavior. Jabedul et al. [16] crosslinked PHA polymer composites for superior thermal stability by reacting formaldehyde with a precursor solution of 4,4′-oxydianiline (ODA) and cyclodextrins (CDs) (α-, β-, and γ-). The material obtained under optimal conditions (ODA:CD molar ratio of 1:0.5, 37% aqueous solution of formaldehyde (formalin)) exhibited good film formability and high thermal stability with two characteristic decomposition phenomena and a high char yield. The CD/PHA composites exhibited inherent flexibility and could act as a carbon source for laser ablation studies. 

The development of biocomposite materials used as adsorbents to remove ions in aqueous media was also the subject of a study performed by Reynoso-Cuevas et al. [17], wherein Valence orange (Citrus sinensis) and Red Delicious apple (Malus Domestica) peels were modified via alkaline treatment, carboxylation and impregnation with zirconium (Zr). The results showed changes in surface area and composition, and most notably an increment in roughness and Zr impregnation of the bioadsorbents. After batch experimentation, the maximum capacity of the materials was determined to be 4.854 and 5.627 mg/g for the orange and apple peel bioadsorbents, respectively, at pH 3.5. The high selectivity of the materials was demonstrated without any significant interferences with the rest of the ionic species (HCO_3_^−^, As(V), SO_4_^2−^, PO_4_^3−^, Cl^−^ and NO_3_^−^) studied. 

Rare earth metals complexed with a polymeric matrix, or so-called polymer–rare earth complexes, possess the combined properties of luminescent rare earth metals and a polymer matrix, being of great significance for potential applications in many fields, such as luminescent therapy probes in vivo, photo-driven catalysts, photoluminescent/electroluminescent polymer films and active layers in solar cells. Chen et al. [18] reported the synthesis, structural analysis and properties of europium- and terbium-based coordination complexes of poly-*N*-isopropyl,*N*-methylacrylamide-stat-*N*,*N*-dimethylacrylamide copolymer, named poly-Eu(III) and poly-Tb(III), respectively. The study of their fluorescence emission characteristics and luminescence lifetime demonstrated that the complexes have coordination interactions between the O and N atoms of the acylamino group with Eu(III) and Tb(III), associated with strong emission peaks at 622 and 545 nm, respectively. The cell viability assessment verified that poly-Eu(III) and poly-Tb(III) can greatly enhance biocompatibility and reduce cell toxicity, showing very promising potential as luminescent probes, especially in the biomedical field.

Most recently, the use of biopolymers as polymeric catalysts and catalyst supports has played an important role in the area of chemical research. Chitin and its derivative chitosan are used in a variety of applications, including as catalysts and catalyst supports. Because of its chemophysical and biological features, such as being hydrophilic, positively charged, biodegradable, non-toxic and biocompatible, chitosan is a recognized biopolymer. Asad et al. [19] synthesized the novel piperidinium dicoumarol via the reaction of 3-formylchromone, 4-hydroxycoumarin and piperidine under chitosan catalyzed solvent-free green conditions, resulting in a short time span, significant yields of products and absence of toxic byproducts or wastes. This protocol can be efficiently applied to a wide variety of aldehydes as well as active methylene compounds in excellent yields. 

An interesting class of polymers includes hydrogels: hydrophilic three-dimensional networks able to accommodate a large amount of water. However, most hydrogels possess uneven crosslinking networks constructed with natural macromolecules, without any effective energy dissipation mechanism preventing possible crack propagation or stress concentration. On this account, *dendrobium officinale enzyme* (DOE) was proposed to improve the structural uniformity and the toughness of chitosan/γ-poly(glutamic acid) (CS/γ-PGA) hydrogel [20]. The results indicated that DOE with various amounts of ingredients can make multiple noncovalent crosslinks with the skeleton network of CS/γ-PGA, significantly changing the self-assembly of CS/γ-PGA/DOE hydrogel to form regular protuberance nanostructures, exhibiting stronger toughness and better behaviors for skin care. 

Given the intensive research on polyurethane, hydroxyl or epoxy groups of vegetable oil have been indicated as functional groups suitable for replacing fossil-based raw materials in the preparation of vegetable oil-based polyols and in the synthesis of polyurethane. Jiang et al. [21] reported the synthesis of soybean oil-based polyurethane (SPU) through the reaction of soybean oil-based polyol with isophorone diisocyanate (IPDI). The properties of SPU films were adjusted by changing the R value (the molar ration of -NCO/-OH) and the film-forming temperature. 

Clay-based hybrid structures with organic polymers and metal oxides, especially bentonite-based composites, have been introduced in recent years as the best multifunctional structures for the retention of toxic metal ions from water supplies [22]. A green ZnO@polynaniline/bentonite composite (G.Zn@PN/BE), synthesized as an enhanced adsorbent for As(V) ions, showed superior physisorption properties (213 mg/g) compared to its components (BE (72.7 mg/g), ZnO (42.3 mg/g) and PN/BE (119.8 mg/g)) or other studied absorbents. 

Although many different agricultural wastes have been proposed as porous adsorbents in pollution management, few activation treatments have been tested and applied to the reuse of olive wastes. Creating a new synthesis route for the realization of a new adsorbent can be time- and energy-consuming, but significant help can be derived from atomistic simulations to initially assess the suitability of a certain adsorbent for capturing a specific compound. Benguerba et al. [23] investigated the adsorption mechanisms of methylene blue (MB) onto olive waste residue (OR) treated with KOH (OR-KOH) and onto an OR-KOH and PEG–silica gel composite (OR-KOH/PEG-SG) at various temperatures by supporting the experimental investigations with Monte Carlo ab initio simulations. The results indicated the possibility of integrating two different methodologies (experimental and theoretical–simulative) as an innovative tool for investigating newly synthesized adsorbents and for determining the energetic interactions that can significantly influence adsorption capacity.

Carbon quantum dots (CDs), as emerging fluorescent materials, have achieved rapid development in optical anti-counterfeiting applications. Shen et al. [24] developed an eco-friendly and simple two-step route to synthesize multi-emission CDs using alkali lignin (AL) and 3-aminophenylboronic acid as the precursor and dopant. According to structure, composition, optical properties and DFT calculations, the multi-emission fluorescence mechanism of NB-CDs was ascribed to the synergistic effect of the core state and the surface-defect state of O, N and B. Among them, doping with B-containing functional groups appeared to be the crucial contribution to the unique multi-fluorescence emissions of NB-CDs. The lignin-derived multiple-emission CDs can be regarded as the next generation of multi-level anti-counterfeiting materials due to their outstanding characteristics of excellent fluorescence stability, high cost-efficiency and renewability.

Over the last few years, researchers have realized that surfactant-based viscoelastic (SBVE) fluids can replace traditional polymeric fracturing fluid composition in the oil industry by mitigating problems arising during and after hydraulic operations. In this respect, SBVE fluid systems entangled with worm-like micellar solutions of cationic surfactant (cetrimonium bromide or CTAB) and counterion inorganic sodium nitrate salt were modified by using ZnO nanoparticle additives with the hypothesis of obtaining fluids with improved rheology [25]. Rheological correlation function models were derived for the synthesized fluids using statistical analysis methods. However, these SBVE fluid systems should be more thoroughly investigated before their on-field implementations considering other technical inherent aspects.

Combining the advantages of photoactive piezoelectric nanomaterials and ferroelectric polymers can effectively solve the problem of decomposing organic dyes. Orudzhev et al. [26] synthesized hybrid polymer–inorganic nanocomposite fiber membranes based on polyvinylidene fluoride (PVDF) and bismuth ferrite (BFO) by electrospinning, emphasizing how the orientation of a magnetic field can determine the anisotropy of magnetic properties in the composite. The results of piezo-photocatalytic experiments showed that under the combined action of ultrasonic treatment and irradiation with both visible and UV light, the reaction rate increased in comparison with photolysis, sonolysis and piezocatalysis. As a result of the study, it can be assumed that the use of BiFeO_3_ as a filler can influence the electroactive phase growth, so leading to an increase in the efficiency of piezocatalysis. The presence of magnetic anisotropy may then be utilized as an additional parameter to modulate photocatalytic characteristics.

As a concluding remark, none of the bioplastics on the market currently satisfy the requirements of full sustainability, and thus a clear follow-up of the most promising studies is necessary to acquire new data for a proper life cycle assessment. In addition, an appropriate policy framework for bio-based, biodegradable and compostable plastics is also needed in order to raise awareness of the sustainable benefits of these special products.

## Data Availability

The data presented in this editorial are available in the correspondent cited articles.
